# Impact of COVID-19 on G20 countries: analysis of economic recession using data mining approaches

**DOI:** 10.1186/s40854-022-00385-y

**Published:** 2022-09-05

**Authors:** Osman Taylan, Abdulaziz S. Alkabaa, Mustafa Tahsin Yılmaz

**Affiliations:** Department of Industrial Engineering, Faculty of Engineering, King Abdulaziz University, P.O. Box 80204, Jeddah, 21589 Saudi Arabia

**Keywords:** Hierarchical clustering, CART, Economic recession, Data mining, COVID-19, G20 countries

## Abstract

The G20 countries are the locomotives of economic growth, representing 64% of the global population and including 4.7 billion inhabitants. As a monetary and market value index, real gross domestic product (GDP) is affected by several factors and reflects the economic development of countries. This study aimed to reveal the hidden economic patterns of G20 countries, study the complexity of related economic factors, and analyze the economic reactions taken by policymakers during the coronavirus disease of 2019 (COVID-19) pandemic recession (2019–2020). In this respect, this study employed data-mining techniques of nonparametric classification tree and hierarchical clustering approaches to consider factors such as GDP/capita, industrial production, government spending, COVID-19 cases/population, patient recovery, COVID-19 death cases, number of hospital beds/1000 people, and percentage of the vaccinated population to identify clusters for G20 countries. The clustering approach can help policymakers measure economic indices in terms of the factors considered to identify the specific focus of influences on economic development. The results exhibited significant findings for the economic effects of the COVID-19 pandemic on G20 countries, splitting them into three clusters by sharing different measurements and patterns (harmonies and variances across G20 countries). A comprehensive statistical analysis was performed to analyze endogenous and exogenous factors. Similarly, the classification and regression tree method was applied to predict the associations between the response and independent factors to split the G-20 countries into different groups and analyze the economic recession. Variables such as GDP per capita and patient recovery of COVID-19 cases with values of $12,012 and 82.8%, respectively, were the most significant factors for clustering the G20 countries, with a correlation coefficient (*R*2) of 91.8%. The results and findings offer some crucial recommendations to handle pandemics in terms of the suggested economic systems by identifying the challenges that the G20 countries have experienced.

## Introduction

Declared a pandemic on March 11, 2020, the coronavirus disease of 2019 (COVID-19) has affected the lives of many people in over 192 countries, as it rapidly spread from China to other countries (Qureshi and ul Rehman A 2020; Ye et al. 2020), leading to large-scale quarantine, isolation, travel restrictions, and social distancing measures. Recent estimates indicate that the global economy has experienced a sharp decline by the end of the following year (IMF 2020a, 2020b; McKibbin and Fernando 2020; OECD 2020a; World-Bank 2020). As one of the most severe pandemics worldwide, COVID-19 will have an impact on firm finance and the cost of capital over the next few decades (Goodell 2020). Due to several factors, there is a great deal of uncertainty regarding the future course and economic consequences of the COVID-19 pandemic (McKibbin and Vines 2020). As reported by the Organization for Economic Co-operation and Development (OECD 2020b), due to the immense drop in sales volumes, logistics problems, and the inability of companies to pay for their suppliers, employees, lenders, and investors, the world has been facing unsurmountable financial issues that cause liquidity problems.

Initially, there was an underestimation of the impact of pandemics on the global economy based on these trends. Consequently, weak steps were taken by the financial prudence that inclines investing less and became evident in the aftermath of the outbreak of the COVID-19 pandemic when the consumption patterns in the global economy changed greatly (Nigmonov and Shams 2021). The failure to establish the necessary cooperation by the G20 countries has caused a sharp increase in the number of consumers who have discontinued their business. The purchasing behavior of consumers has changed, and this has turned into a global crisis, devastating the economy and health sector, ultimately leading to socio-economic changes in the world (Mehta et al. 2020). Therefore, since the collapse of the South Sea Bubble in 1720, global economic activity has suffered the largest collapse caused by the COVID-19 pandemic, a much greater collapse than the global financial crisis in 2008 or the Great Depression in the 1930s (McKibbin and Vines 2020). In addition, in comparison to previous pandemics, COVID-19 is a unique pandemic because it affects not only the population’s health but also their transportation and societal welfare (Sadang 2020). Curfews began suddenly, and people felt as if they were psychologically imprisoned. This has pushed scientists and policymakers to exert a worldwide effort to mitigate the medical, economic, and sociological impacts of the COVID-19 pandemic. Such efforts have turned into multidisciplinary endeavors to find solutions and develop strategies to address essential problems. In such efforts, the most important method is applied in the medical field, in which vaccines have been developed to prevent the virus, and many medicines and medical practices have been launched to cure those suffering and prevent them from spreading the infection to other people (Al-Awadhi et al. 2020; Nour et al. 2020). Another method can be the application of policies to provide social and physical distance and the implication of lockdown to curb and/or confine the transmission of the virus. However, even if such efforts assist in controlling the outbreak in most parts of the world by late 2021, the self-reinforcing dynamics of a recession may prolong the slump until the end of Q2 of 2022. Therefore, other economic efforts should also be exerted to neutralize the negative worldwide economic and sociological effects of the pandemic.

Since its appearance, recent studies have substantially focused on stock markets to analyze the effects of the COVID-19 pandemic, and a large number of studies have been published on stock markets using different approaches (Youssef et al. 2021). However, data on the pandemic are heterogeneous; therefore, the problem was considered from a wider perspective, which requires consideration of some clustering techniques, such as data mining, to analyze the data heterogeneity. In this respect, the dynamics based on temporality and geospatial information were scaled, which could enable the prediction of interconnected dynamics in pandemics, such as the number of hospital beds, COVID-19 cases, deaths, recoveries, percentage of vaccinated population, GDP per capita, industrial production, and the amount of money spent by the governments. In this respect, some stochastic approaches have been applied to scale the dynamical progress during pandemics in countries to code similarities using clustering. Therefore, unsupervised (clustering) models have been extensively proposed to identify and uncover hidden patterns in the data (Li et al. 2021). Compared with supervised (classification) models, clustering models play an essential role in industrial and financial businesses. Kou et al. (2021) propose a novel model for predicting bankruptcy based on payment and transactional variables by selecting the best class that includes the optimal variables.

It is possible to model the forthcoming dynamics of countries based on established clusters together with their similarities (Murali 2020). Accordingly, to slow down the transmission of the pandemic, various dynamic precautions that are substantially based on metadata from cleanliness, continual disinfecting, social distancing rules, and prohibition of public transmission have been applied in various countries (Murali 2020). For this purpose, the search engine tool to collect data from Google Trends was used to measure people’s interest in the COVID-19 outbreak in six countries (Costola et al. 2020). The effect of the lockdown on financial indices and relevant lead lag indications in Italy was observed based on stock indices employed to carry out time-varying analysis. Cavallo (2020) gathered data from credit and debit transactions in the United States to analyze the alterations in consumption expenditure samples due to the COVID-19 pandemic to predict the consumer price index. As can be seen, the effect of the pandemic has been analyzed based on several sectors, varying from stock indices to consumption habits. In such analyses, q-Gaussian distributions have been used to forecast the S&P500 stock index based on COVID-19 projections (Karina et al. 2020).

A large volume of literature has reached conclusions on the effects of the pandemic on stock market dynamics; however, there is still a very limited number of studies that examine the impact of COVID-19 on the aforementioned dynamics leading to the economic recession. In addition, although G20 brings together the leaders of the world’s largest economies (McKibbin and Vines 2020), to the best of our knowledge, no prior study has analyzed the economic recession in the G20 countries; therefore, the focus of the present study is to use data-mining tools to cluster and objectively distinguish G20 countries in terms of their GDP/capita, industrial production, and government spending (Johns Hopkins COVID-19 epidemiological data such as COVID-19 cases per population), COVID-19 deaths per COVID-19 cases, COVID-19 recovery per number of COVID-19 cases, COVID-19 vaccinations per population, and hospital beds per 1000 people.

Although the economic activities for investing in public health in the G20 countries differ, this study explored practical evidence that significant economic benefits can be achieved by improving health conditions in the G20 countries and many other countries outside the G20 group. In addition, this study sheds light on significant gaps in the interconnected dynamics of the economy, and the findings can be handled by future researchers interested in the discipline. The objectives and novelty of this study are as follows:

- To use a data capture framework for integrating data records on GDP/capita, industrial production, and government spending for consecutive economic decision making
- To suggest a data-mining (DM) tool for generating a dynamic clustering map with a resilient data-driven method that can determine the reactions of G20 countries based on the captured data
- To explain how clustered G20 countries have reacted to this pandemic concerning the variables mentioned earlier and the reasons for specific countries by focusing on specific economic variables
- To suggest a new, particularly cogitated clustering algorithm that decision-makers such as medical doctors and administrators of the health industry, economy and finance authorities, politicians, and sociologists can use to identify the possible economic impacts of the COVID-19 pandemic in different G20 countries
- To present and discuss how policymakers should take steps for economic reforms to improve GDP and public health and maintain the production growth rate during the pandemic

## Literature review

Throughout the world, there have been many consequences of the huge outbreak of new coronavirus COVID-19, which has affected several areas, including health care, the economy, transportation, and many others. The fact is that there are no escaping points that the G-20 countries will undergo this metamorphosis (Atayah et al. 2021); therefore, it is unavoidable that the G20 countries have had the greatest share of the economic recession in the world due to the pandemic, given that the G20 countries constitute approximately 60% of the land area of the world, 66.7% of the world population, and more than 90% of the GDP (Lin et al. 2018). These countries have complex economic systems that deeply affect their economic and social development. Indeed, exploring the patterns of the G20 countries in terms of economic factors can reveal the complexity of their economic systems and provide support toward policy decisions, specifically when dealing with economic crises such as the COVID-19 pandemic. In addition, owing to the complexity of human reactions that respond to changing social environments, the endeavors to infer economic data are generally complex and challenging to identify clusters and provide rational explanations (Li et al. 2021).

An extensive number of studies have reported the effect of the COVID-19 pandemic on stock markets. Using data from the top 20 countries affected by the outbreak and the countries that reported the most deaths during the outbreak, Salisu and Vo (2020) investigated whether health news obtained through Google searches could be used to predict stock returns. Using a panel test applied to the Chinese stock market by considering company-specific features, Al-Awadhi et al. (2020) investigated the effect of COVID-19 on the Chinese stock market. Sharif et al. (2020) investigated the time–frequency interactions between oil prices, the COVID-19 outbreak, financial instability, geopolitical tensions, and the stock market in the United States using wavelet-based Granger causality and coherence wavelet tests. It has been demonstrated that the term “corona” affects stock behavior during pandemics as Corbet et al. (2021) have used the DCC-GARCH approach to study the relevant impacts. Using quantile regression, Azimli (2020) examined the impact of the COVID-19 pandemic on the level and form of financial dependence in the United States and found a higher degree of dependence in the financial sector when market returns and portfolios were positive in higher percentages. With regard to econometric models, Liu et al. (2020) and Khan et al. (2020) studied the short-term stock market indices of major affected countries in response to the coronavirus outbreak. Including COVID-19 to the list of negative events for stock markets, Topcu and Gulal (2020) discussed the effects on real oil prices and foreign exchange rate fluctuations caused by COVID-19. Some studies have reported stock market volatility in emerging markets in the Middle East, South America, and Central and Eastern Europe (Anser et al. 2021; Salisu and Obiora 2021). Other studies have mainly focused on the impact of the COVID‑19 outbreak on Chinese‑listed tourism stocks (Wu et al. 2021), investigation of macroeconomic parameters of Montenegro using a Bayesian VARX approach (Djurovic et al. 2020) and prevention of crash in stock market as affected by the economic policy uncertainty during the pandemic (Dai et al. 2021).

It has been documented that COVID-19 has a negative impact on exchange rate returns, firm values, and stock market volatility in recent studies (Ali et al. 2020; Dawson 2020; Iyke 2020; Shen et al. 2020). Furthermore, the literature presented here focuses on observing macroeconomic instability caused by the COVID-19 pandemic in different financial scenarios. Taking a closer look at the existing literature, we found that the question of whether COVID-19 had an impact on the economic recession in the G20 countries in terms of other interconnected dynamics remains unanswered. However, G20 countries, based on economic and other dynamic parameters in their ability to handle and stabilize the economy during COVID-19, should be explored. In view of this, we argue here that there is an urgent need for investigation to reveal the relationship between the interconnected dynamics to contribute to the efforts that all countries should exert. As was the case in 2008–09, it is important for the G20 to lead the way in cooperation, just as it was done in 2008–09 as the G20 brings together the largest economies in the world (McKibbin and Vines 2020).

As a result, this study appears to be the first attempt in terms of conducting exploratory research to pinpoint the impact of COVID-19 on the financial performance of G-20 countries by assessing the relationship between interconnected dynamics. Our hypothesis is that the G-20 countries have the largest economies in the world, which means they have large firms and infrastructure capable of deterring the spread of this pandemic. The aim of this study is, therefore, to answer the question of how the G20 countries are performing financially during the pandemic. It thus presents an emerging research question of how interconnected dynamics would affect the hidden pattern of G20 countries to unravel the complexity of parameters, the proposed primary development pathways, and relevant policy decisions. The remainder of this paper is organized as follows. The following section reviews the data collection and econometric model development. The next section analyzes the clustering of G20 countries using unsupervised machine learning tools for clustering and the classification and regression tree (CART) method. Finally, we present results and discussion, followed by a list of references.

## Data and methodology

### Data collection and econometric model development

The proposed clustering method is based on the last data records taken on the 17th of April, 2021, and gathered for all G20 countries: Argentina (ARG), Australia (AUS), Brazil (BRA), Canada (CAN), China (CHN), European Union (EU) (27 countries), France (FRA), Germany (DEU), India (IND), Indonesia (IDN), Italy (ITA), Japan (JPN), Mexico (MEX), Russia (RUS), Saudi Arabia (SAU), South Africa (ZAF), South Korea (KOR), Turkey (TUR), the United Kingdom (GBR), and the United States (USA). The datasets were obtained from the World Bank, World Health Organization (WHO), OECD, GitHub data repository, and trading economics. The following predictive and response variables were considered for the econometric model in this study: country code and statistical analysis.

The model can be defined in the Eq. 1 as follows:

1 $$RGDP_{it} = \beta_{0} + \beta_{1} GDPC_{it} + \beta_{2} {\text{IP}}_{it} + \beta_{3} GSOP_{it} + \beta_{4} RTC_{it} + \beta_{5} DC_{it} + \beta_{6} HB_{it} + \beta_{7} VP_{it} + exogenous\;factors + \mu_{it} .$$

The variables of the econometric model were classified into endogenous and exogenous factors to determine their significance and robustness and their influence on the GDP (economic growth) in the G20 countries. A factor can be endogenous in some models and/or exogenous in others (Azimli 2020), which may occur when one model serves as a component of a broader model. Thus, Kou et al. (2021) developed a model to predict bankruptcy for enterprises where no financial statements are available. They evaluated the predictive power of payment and transactional data-based variables and removed noisy and redundant factors for bankruptcy prediction by comparing the classification performance of the models. The optimum-seeking method was found to be powerful for the optimal feature subset and was guaranteed to find the global optimal solution.

Table 1 lists the endogenous and exogenous factors and their codes that were selected for this study.

**Table 1:** Variables, descriptions, and sources of the econometric model

Variables type	Variables’ name	Variables’ code	Sources
Response	Real Gross Domestic Product	RGDP	
	**Endogenous factors**		
Predictors	GDP/capita,	GDP	Ali et al. (2020)
	Industrial Production	IP	Khan et al. (2020)
	Government Spending Million $ for cases COVID-19/pop	GS	WHO (2021)
	Cases COVID-19 (per population) %	CC	Al-Awadhi et al. (2020)
	Recovery /Total COVID-19 cases	RT	WHO (2021)
	Death /COVID-19 cases	DC	Murali (2020)
	Hospital beds (per 1,000 people)	HB	WHO (2021)
	Vaccinated per hundred people %	VP	WHO (2021)
	**Exogenous factors**		
	Logistic problems	LP	Atayah et al. (2021)
	The emission of money	EM	World-Bank (2020)
	Governments’ microeconomic policy	MP	Dai et al. (2021)
	Supply and demand management	SD	World-Bank (2020)

The dataset reported in Table 2 is related to the endogenous factors. Additionally, Table 3 shows the mean, standard deviation, minimum, maximum, and median of the endogenous factors for the G20 countries. Large standard deviations are observed for the factors related to observations, as seen in Table 3, implying that there are considerable variations among the G20 countries’ statistics for the considered variables. For some countries, quantitative data of endogenous and qualitative data on exogenous factors were unavailable for some periods due to irregular measurements of factors during COVID-19. Many sources have been investigated to compensate for the scarcity of data. Out of the 19 G20 countries, the state members of the European Union were considered as a whole; hence, data were collected for 45 countries according to the essential criteria presented below. G20 countries collectively produce about 85% of global gross domestic product (GDP), 80% of global CO_2_, 75% of world trade, and 70% of global plastic waste, given that they host two-thirds of the world’s population, although the majority of the population is aged. Furthermore, the G20 countries are responsible for two-thirds of external transnational investment activities and contribute to efforts to develop international assistance, being the funding source for three-quarters of worldwide money orders (OECD-2019 report). The CART method was employed to partition the data according to the relationships between the predictors (only endogenous factors) and response (RGDP) to create a classification regression tree. As given in the Eq. 1, seven endogenous factors, four exogenous factors, and one response variable were considered in developing the model. Quantitative data for endogenous factors were available; hence, the response variable was obtained as the result (class) of the clustering obtained from the hierarchical clustering technique for grouping the G20 countries. Although the effects of the exogenous factors are known and clearly observable, they were not considered in the model results because of their qualitative characteristics and other limitations. These kinds of factors can be defined by linguistic terms such as very high, high, average, low, and very low, for which fuzzy logic can be used for quantification. For instance, the effects of logistics and supply problems of goods and services are in this category; high and irrepressible inflation has been observed in all countries because of these factors. Li et al. (2021) stated that financial data are social data and are generally dominated by multiple intricate variables that can be easily influenced by social life factors and even evolve over time. In contrast, the effects of monetary emissions printed by governments to reduce the impact of unemployment also caused high inflation.

**Table 2:** G20 countries and the factors studied and the related datasets

Country	Country code	Population (Million)	COVID-19 cases	COVID-19 deaths	COVID-19 recovery	GDP/capita ($)	Industrial production*	Government spending million $	Vaccinated/population %	Hospital beds (per 1,000 people)
Argentina	ARG	44.94	203,3060	50,432	1,838,291	9,729	4.9	1,007.52	13.35	4.99
Australia	AUS	25.68	28,905	909	25,486	57,071	− 2	80,891.85	4.45	3.84
Brazil	BRA	210	992,198,1	240,940	8,883,191	11,122	8.2	31,145.93	15.74	2.09
Canada	CAN	37.78	826,924	21,311	774,511	51,589	− 4.98	336,129.4	24.82	2.52
China	CHN	1,400	101,576	4,636	84,602	8,254	7.3	3,691,683	12.91	4.31
European Union	EU	342	171,546,69	155,245	8,569,886	41,388	− 0.8	715,430	24.25	4.71
France	FRA	66.98	3,489,129	82,812	244,238	44,317	− 3	167,209.5	25.06	5.91
Germany	DEU	83.2	2,350,399	66,164	2,013,573	47,628	− 1	206,810	25.12	8
India	IND	1,312	10,937,320	155,913	10,644,858	2,169	1	49,420	8.63	0.53
Indonesia	IDN	270	1,233,959	33,596	1,047,676	4,451	2	20,310	6.04	1.04
Italy	ITA	60.36	2,739,591	94,171	2,268,253	35,614	− 2	95,926.53	23.86	3.14
Japan	JPN	126	419,015	7,236	391,867	49,188	− 2.6	1,094,000	1.49	12.98
Mexico	MEX	127	2,004,575	175,986	1,563,992	10,276	− 2.1	104,465.9	9.79	0.98
Russia	RUS	147	4,112,151	81,446	3,642,582	12,012	− 2.5	57,710	10.49	7.12
Saudi Arabia	SAU	34.22	373,368	6,441	364,646	20,542	− 10	47,930.01	19.43	2.24
South Africa	ZAF	58.8	1,494,119	48,313	1,399,829	7,346	1.8	43,504.75	0.51	2.3
South Korea	KOR	51.78	84,946	1,538	75,360	28,606	3.4	71,000	2.85	12.43
Turkey	TUR	83.15	2,602,034	27,652	792,395	14,999	9	8,442.616	23.19	2.85
United Kingdom	GBR	66.65	4,228,998	118,195	3,315,934	43,688	− 3.3	145,392.9	61.64	2.46
United States	USA	329	27,756,624	488,081	12,965,542	55,809	− 1.8	3,317,000	60.50	2.87

^*^Industrial production attributes the product of industrial foundations, covering some sectors such as mining, manufacturing, electricity, etc. This indicator is measured as an index based on a reference period that expresses change in the volume of production output

**Table 3:** Statistics for the considered variables

Statistics	COVID-19 cases	COVID-19 deaths	COVID-19 recovery	GDP/ capita ($)	Industrial production*	Government spending million $	Vaccinated/population	Hospital beds (per 1,000 people)
Mean	4,694,667	93,051	3,045,336	27,790	0.08	514,271	0.19	4.37
Std. Dev	6,964,866	114,831	3,921,762	19,479	4.70	1,058,752	0.17	3.46
Min	28,905	909	25,486	2,169	− 10	1,008	0.01	0.53
Max	27,756,624	488,081	12,965,542	57,071	9	3,691,683	0.62	12.98
Median	2,191,730	58,298	1,481,911	24,574	− 1.4	88,409	0.15	3.01

An important parameter influencing GDP is industrial manufacturing in sectors such as mining, production, and electricity. During the pandemic, industrial manufacturing and supply to markets were almost zero, or even negative. This created a huge gap in demand after the pandemic. When the economies restarted to accelerate, demand increased tremendously, which could not be met, and problems arising due to logistic issues could not be solved. This indicator was measured using an index based on the reference period.

**Fig. 1:**
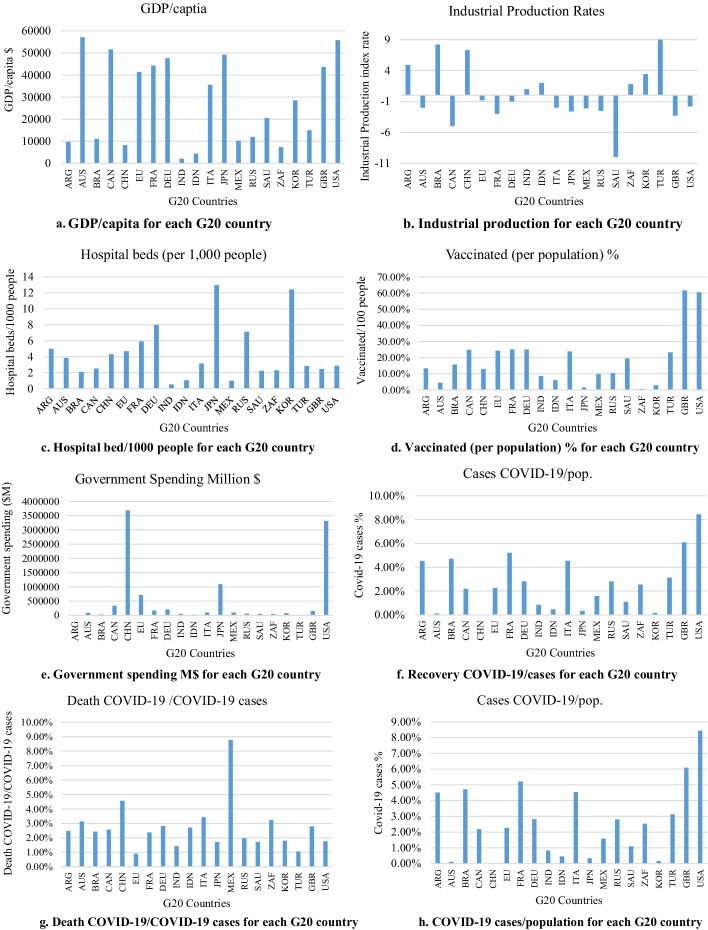
**a** GDP/capita for each G20 country. **b** Industrial production for each G20 country. c Hospital bed/1000 people for each G20 country. d. Vaccinated (per population) % for each G20 country. **e** Government spending M$ for each G20 country. **f** Recovery COVID-19/cases for each G20 country. **g** Death COVID-19/COVID-19 cases for each G20 country. **h** COVID-19 cases/population for each G20 country

The charts shown in Fig. 1a–h were used to compare each country in the G20 with respect to the factors. Based on these charts, these countries demonstrated inconsistent patterns. For example, Fig. 1a shows that Japan had approximately $ 49,000 GDP/capita, whereas India possessed the lowest value of around $2,000 GDP/capita. Among the G20 countries, the highest percentage of deaths due to COVID-19 was observed in Mexico (Fig. 1g). When the G20 countries were compared in terms of COVID-19 vaccination per 100 people, the United States and the United Kingdom had by far the greatest percentage, showing that they vaccinated more than half of their population (Fig. 1d). It is remarkable to observe that Korea and Japan, as the far east countries of Asia, had the highest capacity of hospital beds at a ratio of 12 hospital beds/1000 people (Fig. 1c). However, these countries had the lowest rate of COVID-19 vaccinated/100 people (Fig. 1d) although they had high GDP/capita.

The correlation between a combination of two variables was computed using the Spearman’s rank correlation coefficient (Eq. 2):

2 $$\rho = 1 - \frac{{6\mathop \sum \nolimits_{i = 1}^{n} d_{i}^{2} }}{{n\left( {n^{2} - 1} \right)}},$$

Here *n* denotes the number of observations, *ρ* shows the Spearman’s rank correlation coefficient, and *d*_*i*_ presents the difference between two ranks of each observation. Table 2 also shows the underlying dynamics of the G20 countries, as indicated by several indicators. Table 3 illustrates that the mean GDP per capita is 27,790 $ for G20 countries. The standard deviation of GDP per capita is 19,479$, which is high because of the populations of China and India. Table 3 also shows the maximum (57,071$) and minimum (2,169$) GDP values. The second important indicator of economic recession is the industrial production of the G20 countries. As seen in Table 3, the average increase is 0.08%, the standard deviation is 4.7%, and the maximum rate of increase is 9%; however, industrial production declined by 10% in some countries by 2020. Alongside a significant, coincidental slowdown in consumption, finance, and labor markets during the pandemic, a significant shift in people's negative economic sensitivity was observed in the G20 countries. The results of consumer surveys and transaction data for G20 countries are in line with previous findings for many G20 countries, such as the United States and European countries, France, Italy, Spain, and the United Kingdom. The data from the G20 countries tend to suggest a larger share of the impact of lockdowns compared to other available evidence, as shown in Tables 2, 3, and 4, although data from those countries support the claim that the decline in household spending is partly related to the spread of the virus, independent of mobility restrictions.

**Table 4:** Correlation matrix showing the significance between parameters

Endogenous factors	Endogenous factors	Spearman *ρ*	*p*-value
Industrial Production	GDP/capita	− 0.537	0.0146*
Government Spending Million $	GDP/capita	0.585	0.0067*
Government Spending Million $	Industrial Production	− 0.4054	0.0762
Cases COVID-19/pop	GDP/capita	0.197	0.4052
Cases COVID-19/pop	Industrial Production	− 0.0842	0.724
Cases COVID-19/pop	Government Spending Million $	− 0.0677	0.7768
Recovery COVID-19/Cases COVID-19	GDP/capita	− 0.2586	0.2709
Recovery COVID-19/Cases COVID-19	Industrial Production	− 0.0812	0.7335
Recovery COVID-19/Cases COVID-19	Government Spending Million $	− 0.3203	0.1686
Recovery COVID-19/Cases COVID-19	Cases COVID-19/pop	− 0.4105	0.0722
Death COVID-19 /COVID-19 cases	GDP/capita	− 0.1053	0.6587
Death COVID-19 /COVID-19 cases	Industrial Production	− 0.0256	0.9148
Death COVID-19 /COVID-19 cases	Government Spending Million $	0.1053	0.6587
Death COVID-19 /COVID-19 cases	Cases COVID-19/pop	− 0.0707	0.7672
Death COVID-19 /COVID-19 cases	Recovery COVID-19/Cases COVID-19	− 0.0692	0.772
Hospital beds (per 1,000 people)	GDP/capita	0.4767	0.0336*
Hospital beds (per 1,000 people)	Industrial Production	− 0.0459	0.8477
Hospital beds (per 1,000 people)	Government Spending Million $	0.3805	0.098
Hospital beds (per 1,000 people)	Cases COVID-19/pop	− 0.0406	0.865
Hospital beds (per 1,000 people)	Recovery COVID-19/Cases COVID-19	− 0.1474	0.5352
Hospital beds (per 1,000 people)	Death COVID-19 /COVID-19 cases	− 0.1353	0.5694
Vaccinated (per population) %	GDP/capita	0.4541	0.0443*
Vaccinated (per population) %	Industrial Production	− 0.2843	0.2244
Vaccinated (per population) %	Government Spending Million $	0.3519	0.1281
Vaccinated (per population) %	Cases COVID-19/pop	0.7128	0.0004*
Vaccinated (per population) %	Recovery COVID-19/Cases COVID-19	− 0.5053	0.0231*
Vaccinated (per population) %	Death COVID-19 /COVID-19 cases	− 0.0977	0.6818
Vaccinated (per population) %	Hospital beds (per 1,000 people)	0.0346	0.8849

*Correlation is significant (*p*-value < 0.05)

Table 4 reports the correlation matrix and *p*-value (Spearman *ρ*) for testing the significance of each correlation. Furthermore, it shows the correlation matrix, hence the correlations between industrial production and GDP/capita, Government Spending Million $ and GDP/capita, hospital beds (per 1,000 people) and GDP/capita, vaccinated per hundred people percentage and GDP/capita, vaccinated per hundred people percentage and cases of COVID-19/population, and vaccinated per hundred people percentage and recovery COVID-19/cases are significant because the *p* < 0.05. In contrast, the correlation between recovery COVID-19/cases and cases of COVID-19/population is 0.0722. The correlation between Government Spending Million $ and Industrial Production is 0.0762 and close to the significant *p*-value.

**Fig. 2:**
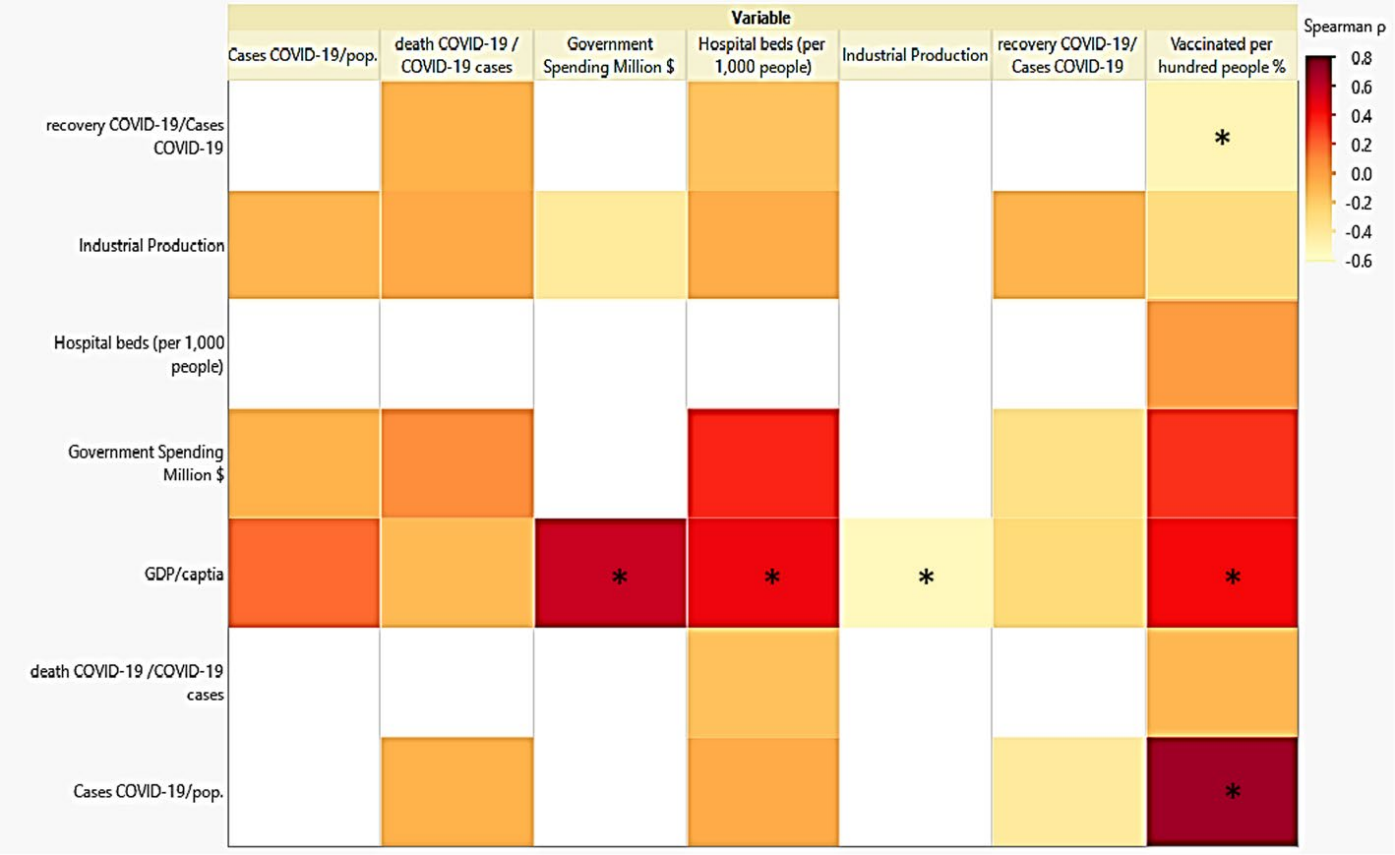
Correlation heatmap of all studied variables (*denotes sig. correlation)

The heatmap presented in Fig. 2 and the findings presented in Table 4 depict the highest association between the rate of vaccinated people and cases of COVID-19/population, with a positive and significant correlation coefficient (Spearman *ρ*-value of 71.28 and *p*-value = 0.0004). This indicates that as the number of COVID-19 cases per population increased, the percentage of vaccinated people also increased. In addition,, it was clearly observed that for countries with high GDP/capita, the number of vaccinated people also increased. This correlation may be related to the investments in public health that promoted high GDP/capita, which agrees with the study of Raghupathi and Raghupathi (2020), who showed that health expenditures have a positive influence on GDP (economic performance). Thus, policymakers can infer that investing in various healthcare activities can directly and indirectly improve income, GDP, and productivity. In addition, it is essential to mention that healthy people and society can be very productive in all industries and educational institutions, showing that public health can be a significant factor in a reliable economy.

It is also noteworthy to state here that the death COVID-19/COVID-19 cases was the only variable not associated with any variables (Table 4). In contrast, there were also negative correlations between industrial production and GDP/capita (ρ =  − 53.7% and *p*-value = 0.0146) and between vaccinated per hundred people % and recovery COVID-19/cases COVID-19 (ρ =  − 50.53% and *p*-value = 0.0231). These negative correlations could be attributed to the shutdown of many industries and low trading and logistics owing to the COVID-19 pandemic. Moreover, despite a high percentage of people being vaccinated against the COVID-19 virus and its variants, public misconduct is thought to lead to more COVID-19 cases.

### Clustering G20 countries

An unsupervised machine learning tool is considered here for clustering (grouping) the G20 countries to reveal how they react to the COVID-19 pandemic in terms of the studied variables. Specifically, a multivariate technique of clustering G20 countries groups countries based on shared values across all variables. This clustering can reveal the clumping structure of G20 countries based on their reactions to the COVID-19 related economic recession. Moreover, clustering provides significant implications in the G20 countries that can help policymakers measure their economic health in terms of the studied variables (benchmarking), identify the variables in each cluster that are more important than others, and explain why countries’ policymakers in a cluster focus on specific variables and their contribution to the economy. Therefore, clustering can be considered an additional method for achieving economic objectives.

This study used the hierarchical clustering technique to group the G20 countries. The method begins by treating each country (observation) as a single cluster. Then, at each step, two close clusters are grouped into a single cluster in terms of the distance or closeness between clusters. These clustering steps can be represented as a tree called a dendrogram. Ward’s minimum variance was used to measure distance. The distance in Ward’s method was computed by taking the sum of squares between two clusters in the analysis of variance (ANOVA) across all variables. At each iteration, the within-cluster sum of squares was taken as the minimum against all partitions procurable by combining two clusters from the previous generation. Ultimately, the method leads to group clusters with few observations and robustly diverges toward generating clusters with an equal number of roundly investigations. More information can be found in the literature (Milligan and Sokol 1980). Ward’s method considers how the sum of squares (distance between two clusters, say A and B) increases when the clusters are grouped. Ward’s method can be computed using Eq. 3:

3 $$D_{AB} = \frac{{\overline{{x_{A} }} - \overline{{x_{B} }}^{2} }}{{\frac{1}{{N_{A} }} - \frac{1}{{N_{B} }}}},$$

**Fig. 3:**
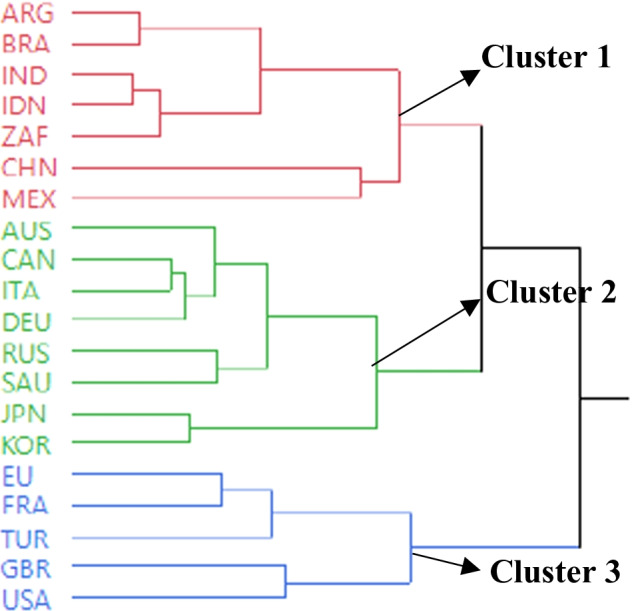
Dendrogram plot for clustering G20 countries

Here D_*AB*_ denotes the distance between clusters A and B, $X$ is the square root of the sum of the squares of the elements of x (the Euclidean length of vector x), and $\overline{{x_{A} }}$ and $\overline{{x_{B} }}$ are the mean vectors for clusters *A* and *B,* respectively, where *N*_*A*_ and *N*_*B*_ represent the number of variables. In this study, a three-cluster solution was found to be optimal because they presented different orientations of COVID-19-related economic and other parameters, which enabled a reasonable interpretation and identification of the countries’ profiles. The dendrogram shows the clustering process by reading it from left to right. The two closest clusters are combined into a single cluster at each step. Figure 3 shows the dendrogram of clustering G20 counties in which the relative distances between clusters were determined, indicating the merging objects (countries) and merging distances (closeness). The numbers of clusters are represented by horizontal coordinates, which decrease from left to right. The vertical coordinate of the point is the margin between the two clusters that participates in creating the determined count of clusters.

### Classification and regression tree method

**Fig. 4:**
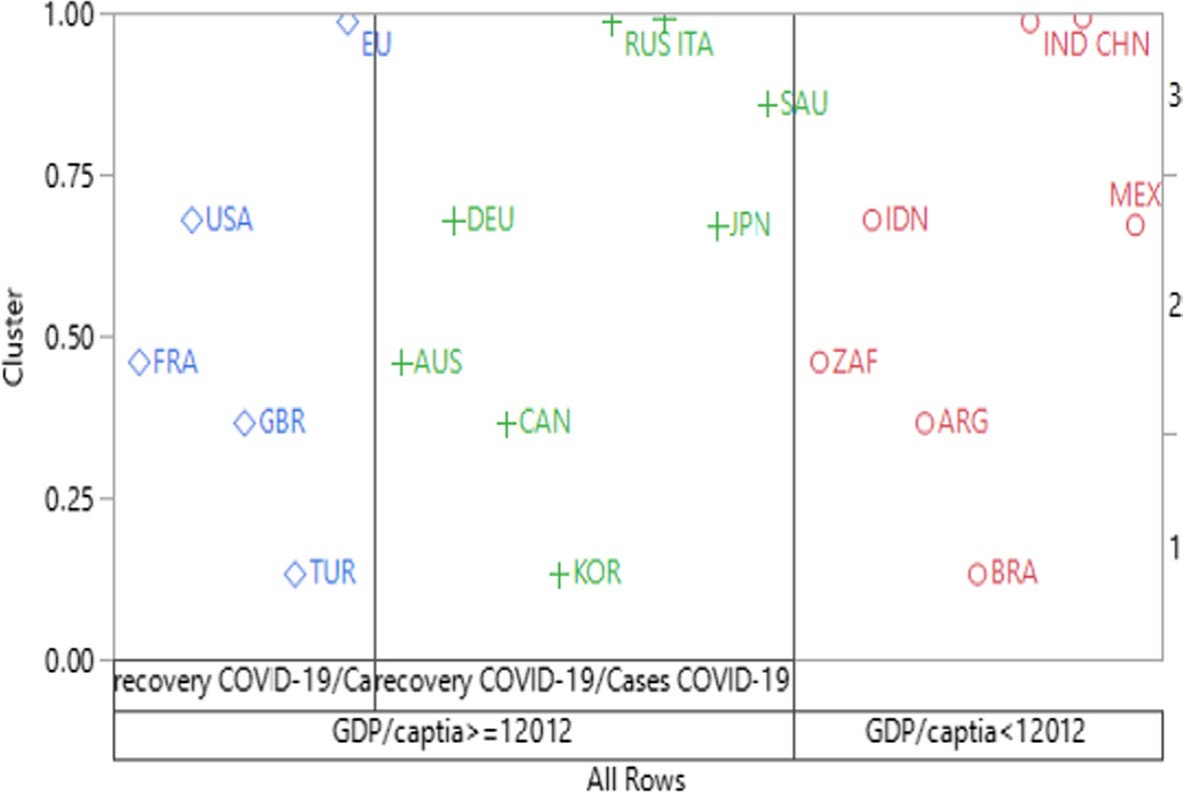
Representation of clustering of G20 countries based on classification regression tree

In this study, the CART method was employed to predict the associations between response (cluster resulting from Ward’s method) and predictor factors. The contribution of CART in this study is the identification of the significant independent variable(s) in the model to split the G-20 countries into different groups. Therefore, this method could help to explore the complex patterns that the G20 countries followed regarding economic and health variables. CART provides significant innovation in nonparametric statistics, data mining, machine learning, and other artificial intelligence approaches (Breiman et al. 1984). It is a partitioning technique and has a recursive property that categorizes the regression tree of continuous data and/or the classification tree of categorical data at each node by employing a set of If Then–Else rules (Timofeev 2004). Wu et al. (2008) stated that CART is one of the top ten most prominent data-mining techniques and has many applications in medical research, biology, different engineering fields, and finance. Likewise, the variables that have direct relevance to our research objective were selected in our study; therefore, we investigated and included these variables that triggered variation in GDP growth. In this study, MATLAB and JMP software were employed to analyze CART, and the results were used for the machine learning approach. RGDP is affected by several parameters, which are principal indicators of the scale of economic activity and economic development, and the variability in RGDP growth is an explanatory response for the world economy. The variation in its slope may represent the underlying growth dynamics or economic recession that occurred in the G20 countries during our study period. The CART partition data were based on the relationship between the predictors and response variables, creating a classification regression tree. More information on recursive partitioning can be found in the literature (Kass and Hawkins 1982; Kass 1980). In modeling CART, the response variable was the clustering results (classes) obtained from the hierarchical clustering technique for grouping G20 countries. The predictors were the eight variables listed in Table 1. The CART model was formulated and is presented in Eq. 1. The resulting classification tree divides the countries into clusters based on the most promising (contributed) variables. Figure 4 shows the proportion of each cluster outcome on the left axis, and the right vertical axis presents the order in which the cluster classes are plotted. Horizontal lines divide each split by the most contributed response variables of GDP/capita and recovery COVID-19/Cases COVID-19 (resulting from the most significant variables in the CART model). The first horizontal line shows the overall proportion of the first plotted response based on the (GDP/capita), while the second horizontal line shows clustering based on the recovery COVID-19/cases COVID-19. Countries in the same cluster are similar with respect to their GDP/capita and COVID-19/cases COVID-19.

**Fig. 5:**
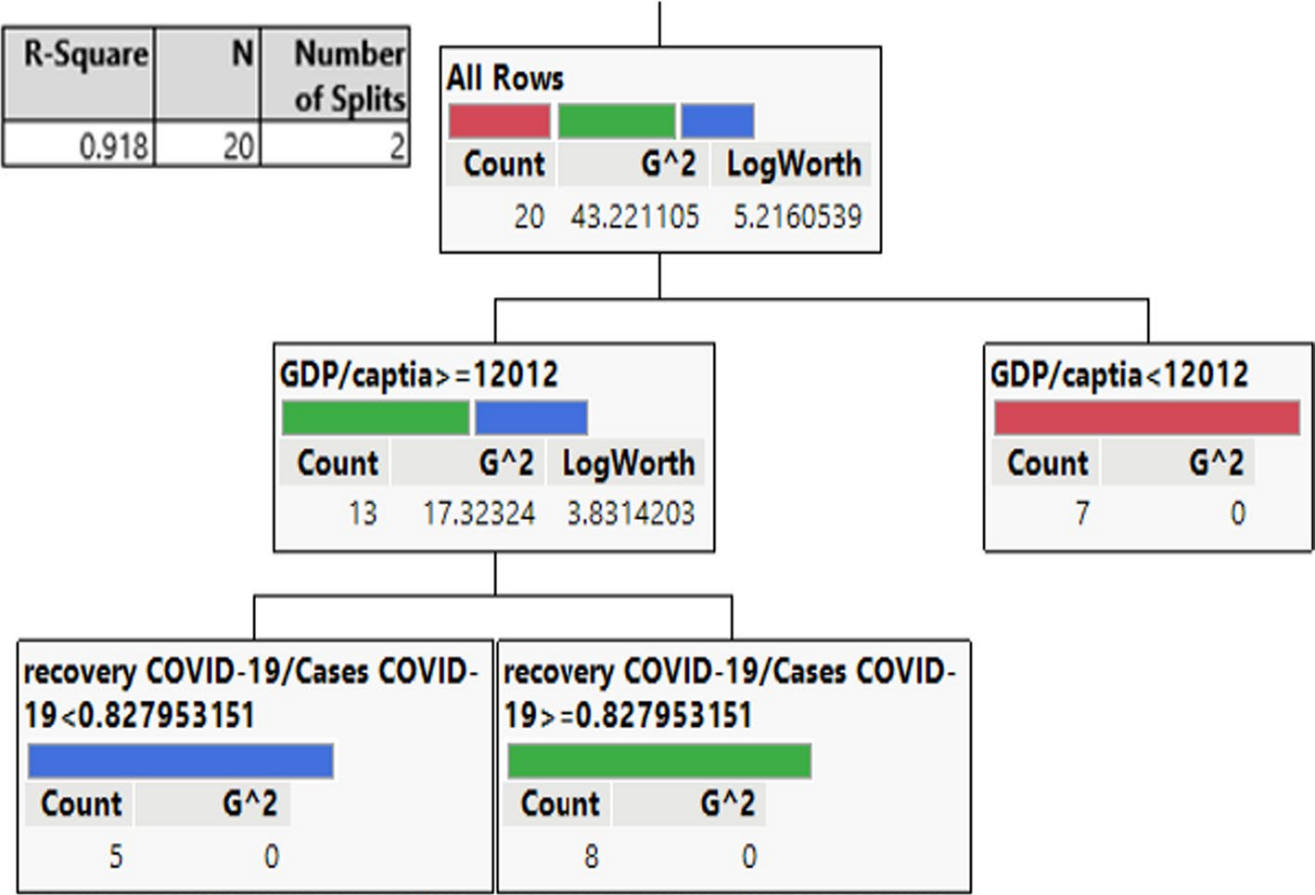
Classification regression tree

Each node shown in Fig. 5 is a relevant predictor that can separate each cluster. The splitting value is expressed as a mathematical inequality rule. For example, when the GDP/capita is ≥ $ 12,012, the cluster contains 13 country codes colored in green and blue with a G^2^ = 17.32. These values represent a fit statistics corresponding to the sum of square and logworth (= 3.83), denoting the optimal split that maximizes the logworth, as shown in Fig. 5. In contrast, when the GDP/capita is < $ 12,012, the cluster includes seven country codes, colored in red. The R^2^ (0.918) value explains the amount of variation in clustering, which accounted for splitting the variables of GDP/capita and recovery COVID-19/cases COVID-19, with their specified values in the CART. Therefore, policymakers in these countries should be keen on stabilizing and controlling their GDP and healthcare. Japan and South Korea were grouped into one cluster. Accordingly, these countries are in the far east of Asia, with the highest number of hospital beds/100 people, and the lowest number of COVID-19 cases. Thus, the implications of such a cluster revealed that healthcare is highly valued in these countries. In contrast, limited attention has been paid to healthcare in the G20 countries, despite the highly related policy insights. Investing in people can have a major effect on all economic activities. Adequate policy responses should involve different areas, including industrial production, the business environment, healthcare, innovation in technologies, and research and development. For instance, to minimize the financial impact of the pandemic, the WTO announced on April 15, 2021, a proposal for vaccination of all laborers in industries to resume production, reduce export constraints, and pause intellectual property rights on COVID-19 vaccines to boost immunizations.

## Results and discussion

Table 5 lists the cluster means for different parameters. Cluster 1 includes seven countries: Argentina, Brazil, India, Indonesia, South Africa, China, and Mexico. Their mean of GDP/capita is 8,543.25, the industrial production is 4.01, and the government spending is 493,747.5. However, the death rate is the highest, indicating that these countries do not curb the influences of the COVID-19 on their economies. A direct correlation is observed between the percentage of people vaccinated per population and the number of deaths. In addition, the number of beds per 1000 people is 2.38 in the countries within this cluster, and this is the lowest rate as compared to those of the other two clusters. It can be inferred that these countries have not stopped industrial production. Specifically, the industrial production of the countries in Cluster 1 ranged from − 2.1 to 8.2. Except for Mexico, all the other countries have exhibited an economic growth in industrial production. Generally, the policymakers of the countries in Cluster 1 have focused on production stability rather than the public health, which could be affiliated with the type of the ruling regimes and its relationship to the economic activities. Moreover, it appears that the production continuation in these countries did not contribute to GDP increase, which is about < 50% than those of the other countries within Clusters 2 and 3.

**Table 5:** Cluster means of important indicators

Variables	Clusters		
	1	2	3
GDP/capita	8,543.25	38,182	47,938
Industrial Production	4.0125	− 2.49778	− 2.7
Government Spending Million $	493,747.5	300,647.5	1,209,867.463
Cases COVID-19 (per population) %	0.0222	0.0181	0.0658
Recovery COVID-19/Cases	0.8095	0.8541	0.4404
Death COVID-19 /COVID-19 cases	0.0333	0.02235	0.0231
Hospital beds (per 1,000 people)	2.3862	6.3311	3.7466
Vaccinated (per population) %	0.1127	0.1519	0.4907

As a member of Cluster 1, Indonesia has the lowest number of COVID-19 deaths; however, the highest number of deaths is in Brazil, which is also in this cluster. As one of the largest economies, China also belongs to Cluster 1, which has the lowest rate of death/COVID-19 cases but the second-highest industrial production rate of 7.3. This could be mainly ascribed to the fact that the Chinese government’s policymakers have applied innovative/expert systems and AI business automation, as well as Internet medicals and advanced technologies such as extensive data analysis, 5G, and cloud computing (Sun et al. 2021) technologies. In G20, South Africa is the only member of the African continent and has the lowest rate (0.51%) of the vaccinated population in all three clusters. According to the WHO’s infections and deaths report issued in July 2021, the most severe infections and deaths due to COVID-19 have been observed in Namibia, Uganda, Zambia, and South Africa. In contrast, India has the lowest rate (0.53) of beds per 1,000 people in the G20 countries and ($2,169) of GDP/capita. In addition, India declared that its GDP at the second quarter (in April 2021) declined by 25.8% with respect to the one at the first quarter; therefore, foreign investors withdrew an estimation of $16 billion from India. This has led to severe concerns, and it has been the worst economic recession in history (Slater 2021). Consequently, policymakers have taken steps to implement economic reform. For example, in November 2021, India’s finance minister announced a new fiscal program worth $35 billion to support industries, agriculture, and exports. Thus, economic certainty is critical; however, it does not generate sectoral heterogeneity during the pandemic. Consequently, every country has been affected differently, revealing how policymakers in these countries deal with their economic objectives.

As shown in Fig. 3, Cluster 2 included the following eight countries: Australia, Canada, Italy, Deutschland, Russia, Saudi Arabia, Japan, and South Korea. This cluster depicts economically developed countries, that is, their GDP/capita is 38,182 (see Table 5); however, industrial production is negative (-2.49). The countries in Cluster 2 have lower government spending (300,647.5), death rate (0.02), and COVID-19 case rate to the population (0.018) than the other G20 countries in Clusters 1 and 3. In Cluster 2, the number of beds per 1000 people and the recovery of COVID-19/cases are 0.85 and 6.33, respectively, which are the highest rates among all clusters. In contrast, a direct negative correlation was observed between the number of beds and number of deaths. This is indicative of direct political consequences, as it shows that economic policymakers have a rationale for investing in health as an additional strategy to achieve their economic goals. Consequently, health is considered an investment that can provide economic returns rather than cost.

Cluster 3 included the European Union, France, Turkey, the United Kingdom, and the United States. This cluster mainly depicts highly industrialized countries. The GDP/capita is 47,938, which is the highest among all the clusters. Except for Turkey, industrial production was negative in these countries. The average industrial production is − 2.7; however, government spending is higher (1,209,867.463) compared to the other two clusters. The only positive industrial production range was recorded in Turkey, which was 9. In this cluster, the death case rate and the number of beds per 1000 people are 0.02 and 3.75, respectively, which seem similar to the values in other clusters. A negative correlation was observed between the number of beds and deaths in Cluster 3. The number of beds in Cluster 3 was almost half that in Cluster 2; however, the number of deaths was very close to that in Cluster 2. The highest number of vaccinations per hundred people is 0.4907 and is in this cluster. The high death rate could be attributed to the fact that the elderly population rate is also high in these countries. In other words, from a policymaking point of view, shutting down industrial production did not drastically reduce or completely halt the percentage of deaths. The industrial production of these countries ranges from − 3.3 to 9. The number of COVID-19 cases/population is 0.018122, which is lower than that of the other clusters, while the recovery rate of COVID-19/cases is 0.85, which is higher than the rates in the other two clusters.

**Fig. 6:**
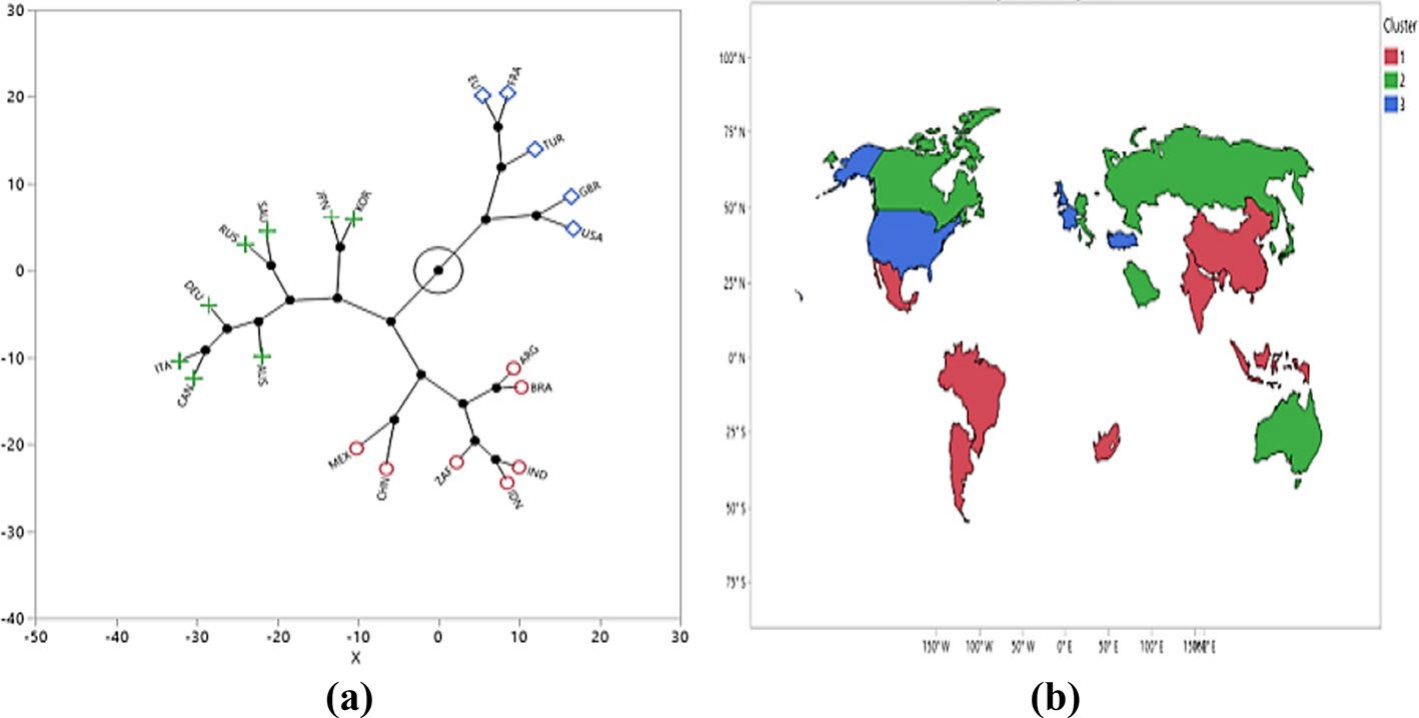
Constellation (**a**) and universe map (**b**) plot for G20 countries

Clustering is a multivariate approach to grouping investigations that share akin valuations away from several variables. A constellation diagram displays the clusters for the ideal (reference) point and the actual measured distance on the same plot. Hence, Fig. 6a shows the ideal point’s location of a constellation diagram predefined universally depending on the shared values chosen for COVID-19 economic and other remaining parameters. Constellation diagrams are helpful for graphically visualizing data to promptly identify standard variables and quantify the disparity between measured and ideal findings. The lines in the constellation diagram represent membership in a cluster. The constellation plot indicates that the three clusters have clear cut-off boundaries and shows the distance between each cluster from the remaining countries in the upper half of the plot and those in the lower half of the plot. Additionally, Li et al. (2021) found that changing social environments and the complexity of human behavior make the distribution of financial data more complex. They developed an integrated approach to detect and optimize financial data clusters and quickly interpret them based on k-means clustering algorithms. In other words, the approaches suggested by Li et al. (2021) and our approach successfully clustered the problems considered and could unravel hidden patterns.

**Fig. 7:**
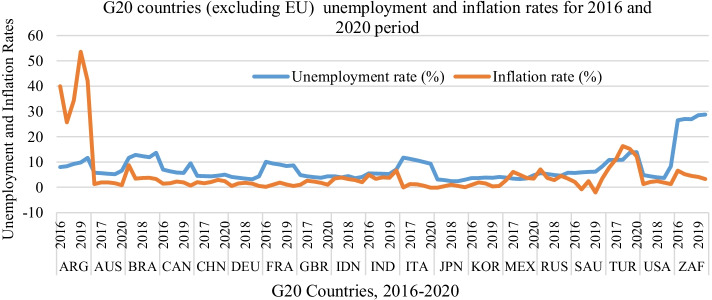
G20 countries unemployment and inflation rates for 2016 to 2020 period

The COVID-19 pandemic-related economic crises can be portrayed by shock waves in terms of supply and demand. The pandemic has caused a profound global socio-economic crisis, with a harmful impact on financial markets, logistics systems, labor, and goods supply chains. Economic activities are restricted or ultimately concluded in many countries; hence, the economy falls into a deeper recession due to supply and demand shocks. This may ultimately lead to stagnation in economies, as described by higher price levels (revenge pricing) and unemployment rates. Remarkably, the structure and power of the negative relationship rely on several elements, such as inflation with respect to its long-dated tendency, the bottom of its extrinsic impact, and political action. The current economic situation permits analysis of the inflation, industrial production, GDP, and unemployment dynamics of countries with great alterations in economic factors, as the world started to gear toward policy intervention for economic recovery from the COVID-19 pandemic. Tables 5 and 6 list the unemployment and inflation rates in the G20 countries, except the European Union, from 2016 to 2020. Figure 7 shows the unemployment rates from 2016 to 2020, indicating that inflation has increased geometrically in Argentina and continues to increase. This trend was also observed in Turkey. The change in other countries is steady; however, a significant fluctuation in the unemployment rate appears in the United States, Brazil, France, Italy, and South Africa. It seems that inflation does not have a distractive effect on economies during 2020, which could be associated with the lockdown implemented by the G20 countries. However, the rising trend of inflation started to disrupt the economies of almost all countries during 2021 and in the first half of 2022.

**Table 6:** G20 countries (excluding EU) unemployment rates

G20 Countries	G20 Countries unemployment rates				
	2016	2017	2018	2019	2020
Argentina	7.97	8.35	9.22	9.84	11.67
Australia	5.71	5.59	5.3	5.16	6.61
Brazil	11.6	12.82	12.33	11.93	13.67
Canada	7	6.34	5.83	5.66	9.48
China	4.5	4.4	4.3	4.6	5
Germany	4.12	3.75	3.38	3.14	4.31
France	10.04	9.41	9.02	8.44	8.62
United Kingdom	4.81	4.33	4	3.74	4.34
Indonesia	4.3	3.88	4.4	3.62	4.11
India	5.51	5.41	5.33	5.27	7.11
Italy	11.69	11.21	10.61	9.95	9.31
Japan	3.1	2.8	2.4	2.4	2.97
South Korea	3.65	3.65	3.82	3.75	4.07
Mexico	3.86	3.42	3.28	3.48	4.71
Russian Federation	5.56	5.21	4.85	4.6	5.73
Saudi Arabia	5.65	5.89	6.04	6.13	8.22
Turkey	10.84	10.82	10.89	13.67	13.92
United States	4.87	4.36	3.9	3.67	8.31
South Africa	26.54	27.04	26.91	28.47	28.74

Owing to the long curfews, the inability of consumers to visit shopping centers freely and the delay in purchasing their needs decreased the demand for goods and services during the pandemic period. This situation has led to high volatility in economic policy. The high emission of money injected by governments to reduce the impact of unemployment also led to high inflation, which created a huge gap during post-pandemic demand as industrial production has declined, and the supply of goods and services has dwindled or nearly ceased. The economy is like a standing ship, and it takes time to regenerate and meet market needs. The demand has increased remarkably after the pandemic, which could not be met immediately, and problems arose due to logistics. This case is remarkable in the United States, which is representative of a developed country and economy, as presented in Table 2. In the United States, the GDP per capita is 55,809, which is the highest after Australia, although the government spending is 3.3 trillion $, and the industrial production is − 1.8. Moreover, the pattern of growth showed a declining trend in the pre-recession period; however, unemployment and inflation rates were low (as shown in Fig. 7).

Table 6 shows the G20 countries unemployment rates excluding the European countries. In 2020, the highest unemployment rate was observed in South Africa (28.74%). The lowest unemployment rate was found in Japan, at 2.97. Table 7 presents the inflation rates of the G20 countries. The highest inflation was observed in Argentina (42%), whereas the lowest was observed in Italy (-0.14%).

**Table 7:** The inflation rates (%) of G20 countries (excluding EU)

G20 Countries	G20 Countries inflation rates (%)				
	2016	2017	2018	2019	2020
Argentina	40	25.7	34.3	53.5	42.0
Australia	1.28	1.95	1.91	1.61	0.85
Brazil	8.74	3.45	3.66	3.73	3.21
Canada	1.43	1.60	2.27	1.95	0.72
China	2.00	1.59	2.07	2.90	2.42
Germany	0.49	1.51	1.73	1.45	0.51
France	0.18	1.03	1.85	1.11	0.48
United Kingdom	1.01	2.56	2.29	1.74	0.99
Indonesia	3.5	3.8	3.3	2.8	2.0
India	4.95	3.33	3.95	3.72	6.62
Italy	− 0.09	1.23	1.14	0.61	− 0.14
Japan	− 0.12	0.47	0.98	0.48	− 0.02
South Korea	0.97	1.94	1.48	0.38	0.54
Russia	7.04	3.68	2.88	4.47	3.38
Saudi Arabia	2.07	− 0.84	2.46	− 2.09	3.45
Turkey	7.78	11.14	16.33	15.18	12.28
United States	1.26	2.13	2.44	1.81	1.23
South Africa	6.59	5.18	4.50	4.12	3.22
Mexico	2.82	6.04	4.90	3.64	3.40

**Fig. 8:**
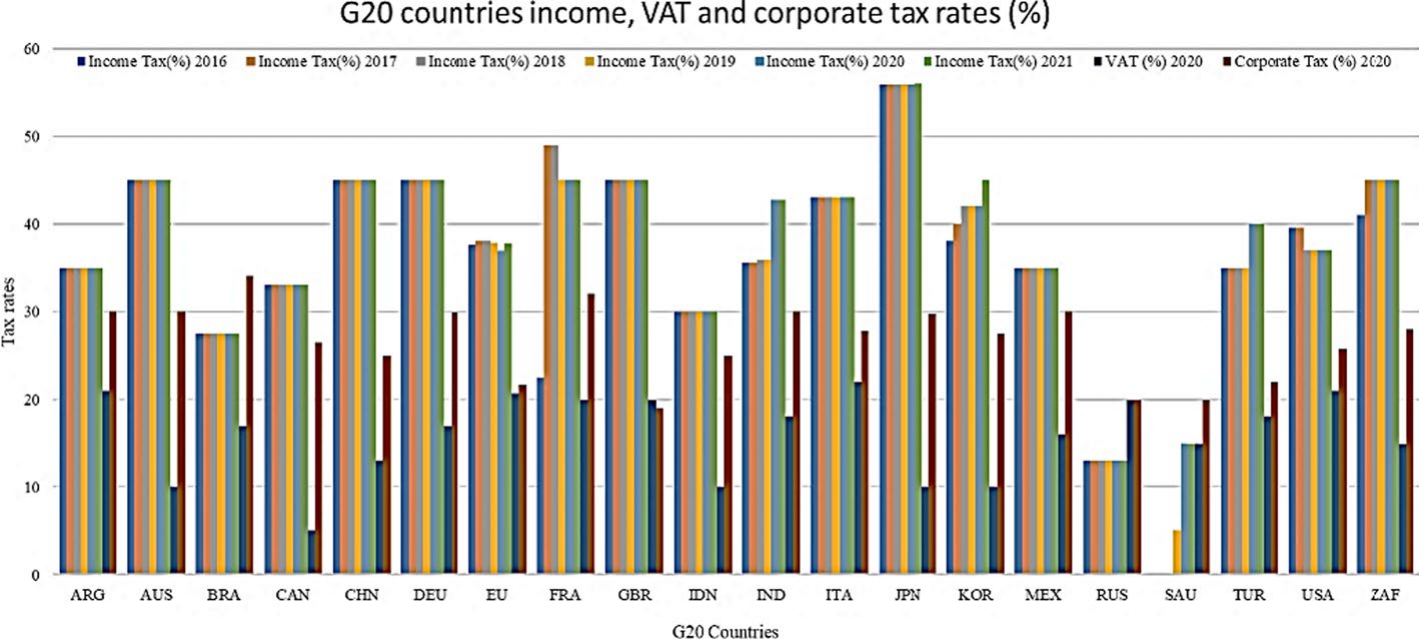
G20 countries income, VAT, and corporate tax rates (%)

**Fig. 9:**
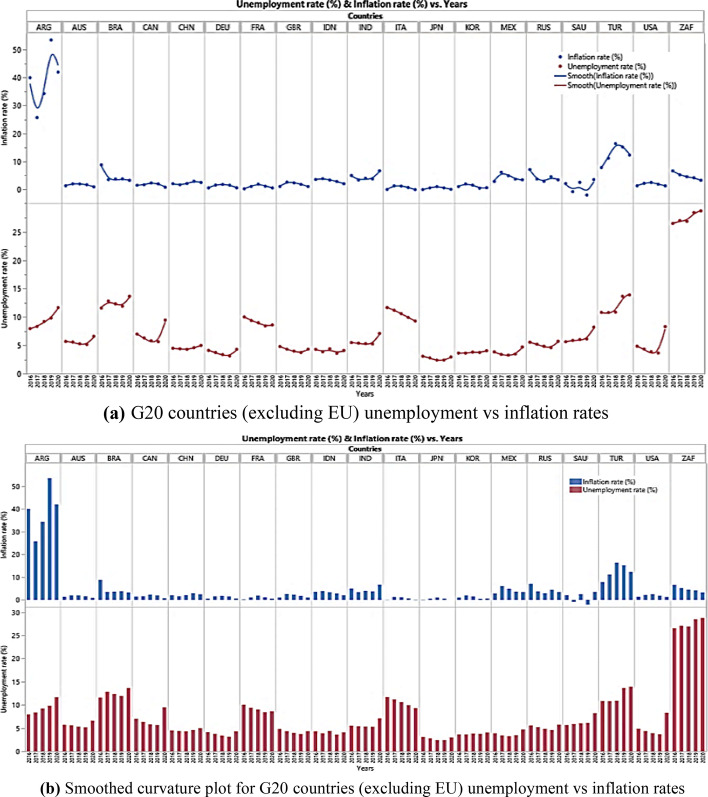
**a** G20 countries (excluding EU) unemployment vs inflation rates. **b** Smoothed curvature plot for G20 countries (excluding EU) unemployment vs inflation rates

**Fig. 10:**
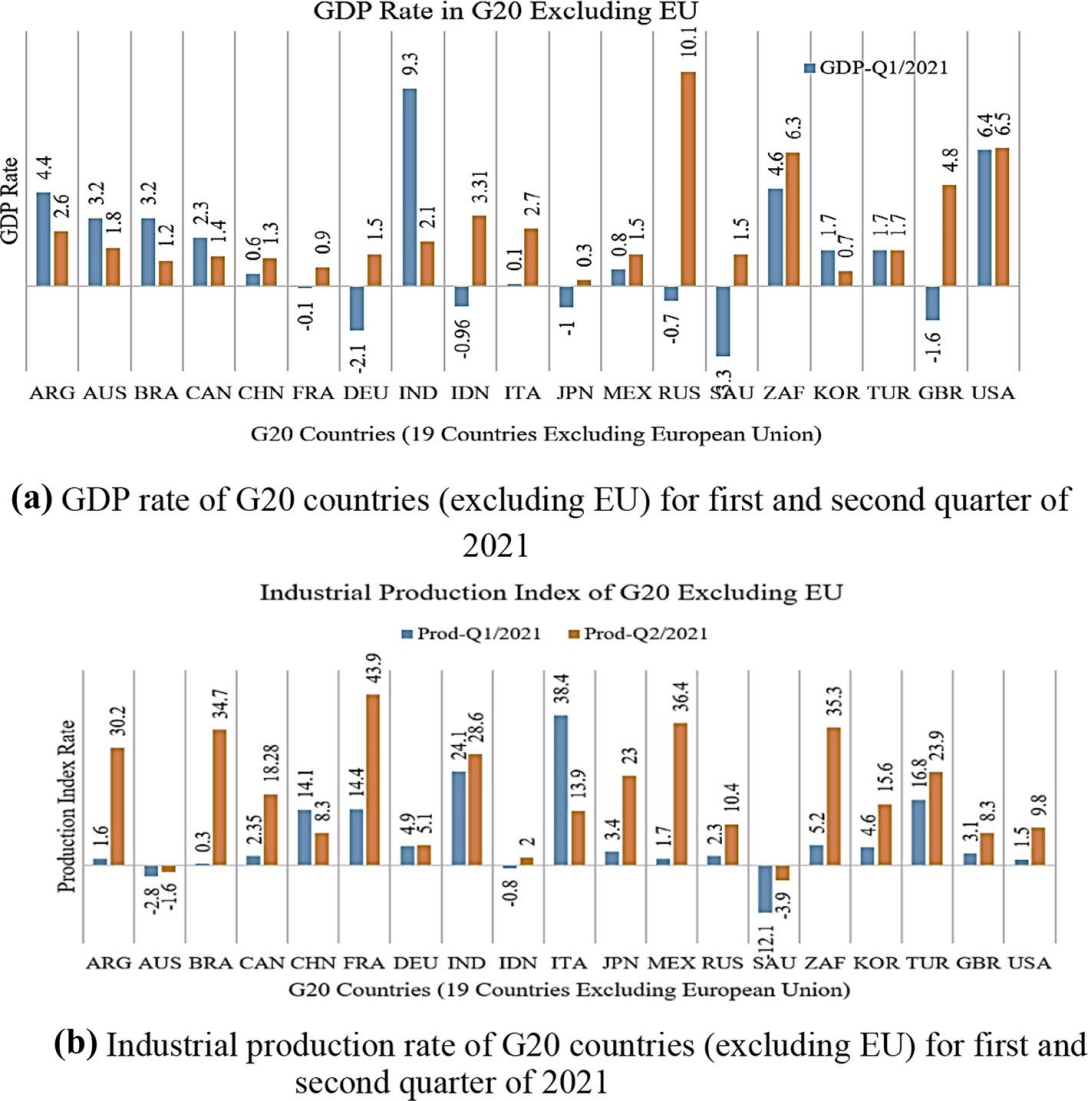
**a** GDP rate of G20 countries (excluding EU) for first and second quarter of 2021. **b** Industrial production rate of G20 countries (excluding EU) for first and second quarter of 2021

Table 8 and Fig. 8 illustrate G20 countries’ income, VAT, and corporate tax rates in percentages. Although the highest income tax was in Japan at 55.97% in 2020, unemployment was the lowest. The highest VAT was observed in Italy (22%), which has a 9.31% unemployment rate. The highest corporate tax was observed in Brazil at a rate of 34%; however, the inflation rate was low (3.21%) and unemployment was high (13.67%). Increasing productivity is crucial and plays an important role in prosperity from a policy perspective because it is the primary driver of RGDP per capita growth and shows improvement in living standards. Figure 9a, b show that the unemployment and inflation relationship is nonlinear and has an inverse relationship, reflecting a negative correlation. During the peak of the pandemic, the unemployment rate increased for almost all G20 countries; however, inflation decreased. Figure 9a, b suggest a short-run trade-off between inflation and unemployment for the G20 countries, aligned with Phillips curve theory. in contrast, as shown in Table 2, the industrial manufacturing of G20 countries has dwindled and/or almost ceased. Similarly, Fig. 10a, b show that the GDP growth depends on several factors.

**Table 8:** G20 countries (excluding EU) income, VAT, and corporate tax rates (%)

G20 Countries	Income tax (%)						VAT (%)	Corporate tax (%)
	2016	2017	2018	2019	2020	2021	2020	2020
EU average	37.58	38.06	38	37.81	36.92	37.77	20.7	21.7
Argentina	35	35	35	35	35	35	21	30
Australia	45	45	45	45	45	45	10	30
Brazil	27.5	27.5	27.5	27.5	27.5	27.5	17	34
Canada	33	33	33	33	33	33	5	26.47
China	45	45	45	45	45	45	13	25
France	22.5	49	49	45	45	45	20	32.02
Germany	45	45	45	45	45	45	17	29.9
India	35.54	35.54	35.88	35.88	42.74	42.74	18	30
Indonesia	30	30	30	30	30	30	10	25
Italy	43	43	43	43	43	43	22	27.81
Japan	55.95	55.95	55.95	55.95	55.95	55.97	10	29.74
South Korea	38	40	42	42	42	45	10	25
Mexico	35	35	35	35	35	35	16	30
Russia	13	13	13	13	13	13	20	20
Saudi Arabia	0	0	0	5	15	15	15	20
South Africa	41	45	45	45	45	45	15	28
Turkey	35	35	35	35	40	40	18	22
United Kingdom	45	45	45	45	45	45	20	19
United States	39.6	39.6	37	37	37	37	5.7	25.77

The curves in Fig. 9a display concave and convex functions, respectively, which also depict dwindled or/and almost ceased RGDP rates during 2020. The concavity pattern showed that inflation was in a declining trend during the pandemic; however, the convexity of the unemployment curve showed an increasing trend in unemployment. A decrease in inflation usually leads to a considerable decrease in unemployment and an increase in the GDP rate. Consequently, unemployment can be said to have enormous societal costs, even though low inflation rates cause minor nuisances. The implications of the negative relationship between unemployment and inflation can be seen in the current monetary policies aimed at raising RGDP and minimizing unstable economic conditions in G20 countries. The monetary policy of some G20 countries is aimed at reducing unemployment; however, this may temporarily increase the inflation rate, which has occurred nowadays. The hike in the prices of crude oil and supply shocks, logistics problems, and unavailability of crucial raw materials have led to increased cost inflation, cooperating with the rising unemployment and dwindling of RGDP. However, this relationship may change in the long run when the price levels of crude oil, energy, and raw materials are adjusted. The decrease in logistics costs may also positively affect GDP in the coming years. For example, US policymakers have emphasized investing in public health and providing time-bound support to families, societies, and firms. Government financial support, if appropriately directed toward investment in industrial manufacturing, can reduce the scarcity of certain advanced products (i.e., chips and circuits) in the market. However, governments seem to focus only on reducing the effects of expanding unemployment (involving additional unemployment benefits), sending immediate stimulus payments of $1,400 to qualified people, delivering immediate support to state and local governments, supplying resources to the vaccination program, and raising grants for academic institutions to reopen. As illustrated in Fig. 9a, b, in general, the trends provide a good understanding of the core dynamics at the system level. In France and Italy, unemployment declined; however, it showed an increasing trend in all the other G20 countries.

The inflation rates did not show an underlying variation in the G20 countries during the peak of the pandemic. However, in the post-pandemic period, very high rates were observed in the prices of goods and services. We developed the following econometric model to estimate the real GDP of the G20 countries based on Eq. 1. We also determined the standard deviation of RGDP growth in the recession periods of the G20 countries as an additional explanatory variable. Table 9 shows the coefficients of the econometric model and t-statistics ratios. Additionally, the *F* ratio and coefficient of determination were 9.9363 and 0.8784, respectively.

$$\begin{aligned} RGDP_{it} & = 4021.7941 + 0.0218 GDP_{it} - 45.113 IP_{it} + 0.0045 GS_{it} \\ & \quad \quad + 1702.66 CC_{it} - 2480.476 RT_{it} - 43598.42 DC_{it} - 83.9508 HB_{it} \\ & \quad \quad + 2171.292 VP_{it} + exogenous\;factors + \mu_{it} \\ \end{aligned}$$

**Table 9:** Coefficient of econometric model and its statistics

Endogenous factors	Estimated parameters	Std Error	t Ratio	Prob >|t|
Intercept	4021.7941	3403.366	1.18	0.2622
GDP/capita	0.0218362	0.048401	0.45	0.6606
Industrial Production	− 45.11372	165.7479	− 0.27	0.7905
Government Spending Million $	0.0045444	0.000654	6.95	< 0.0001*
Cases COVID-19/pop	1702.6641	46,903.62	0.04	0.9717
Recovery COVID-19/Cases COVID-19	− 2480.476	2866.706	− 0.87	0.4054
Death COVID-19 /COVID-19 cases	− 43,598.42	38,586.76	− 1.13	0.2826
Hospital beds (per 1,000 people)	− 83.95083	226.6417	− 0.37	0.7181
Vaccinated per hundred people %	2171.2924	7760.956	0.28	0.7848

^*^p-value < 0.0, signficant variable

The econometric model indicated that endogenous factors are key indicators affecting the economies of the G20 countries, relying on the assumption that producers can expedite production in response to the COVID-19 pandemic. Hence, emerging economies with large manufacturing bases are expected to recover quickly, while weaker manufacturing-based economies are expected to suffer from long-term downward and output contraction trends. Hence, as shown in Table 5, the average industrial production growth is 4.01% for Cluster 1, including Argentina, Brazil, India, Indonesia, South Africa, China, and Mexico. Industrial production has declined by approximately − 2.49% for the countries in Cluster 2, including Australia, Canada, Italy, Deutschland, Russia, Saudi Arabia, Japan, and South Korea. Similarly, the average industrial production declined by about − 2.7% for the countries in Cluster 3, which includes the European Union, France, Turkey, the United Kingdom, and the United States. Our analysis showed that the shift in economic indicators was significantly more prominent in EU countries; the GDP rates, labor market conditions, and vaccination process were less favorable at the beginning of the crisis. Failing to develop adequate and harmonious policies causes economic deviations and risks among EU member states.

Hence, data from the first and second quarters of 2021 for GDP, unemployment, inflation, and industrial production were analyzed. With the shift in economic sentiment during the first two quarters of the year, the impact of the COVID-19 pandemic seems to be reduced; this is especially the case for unemployment-related sentiment. Following the pandemic, unemployment-related searches jumped far beyond those observed during the Great Recession. As shown in Fig. 10a, b, GDP growth depends on many factors. For instance, industrial productivity is the primary driver of prosperity and GDP per capita growth. Therefore, it is crucial from a policy perspective. The bulk of GDP per capita growth in the first and second quarters of 2021 in all G20 countries is growing; the maximum growth appears in France and Mexico, with rates of 43.9% and 36.4%, respectively. However, economic contraction and disruption still appear in Australia and Saudi Arabia, with rates of − 1.6% and − 3.9% in the second quarter of 2021, respectively. With the aging societies of EU countries in the G20, increased productivity will improve living standards and positively affect growing economies. Banerjee et al. (2020) state that the COVID-19 crisis raised uncertainty, caused a decline in corporate investment, and added a strain on corporate liquidity that might further weaken industrial productivity growth in future international trading bans, and logistic problems are not eliminated. It seems that the slowdown in industrial productivity growth is temporary and not structural.

However, announcing the slowdown implications that cause policy concerns or structural problems early. The COVID-19 pandemic might speed up the structural changes triggered and offer several challenges and opportunities for the G20 countries. Lower productivity growth, lower business dynamism, and high correlation may increase the divergence between the most and the least productive firms. The delay in the availability of relevant official statistics, long-term uncertainties in economic bias, and the impact of the COVID-19 pandemic on productivity cannot be definitively determined at this stage because of the exogenous factors presented in Table 1. Additionally, a slowdown in workers' reorganization and government support will result in worsening of labor skills; hence, the destruction of jobs can reduce productivity in the long run.

**Fig. 11:**
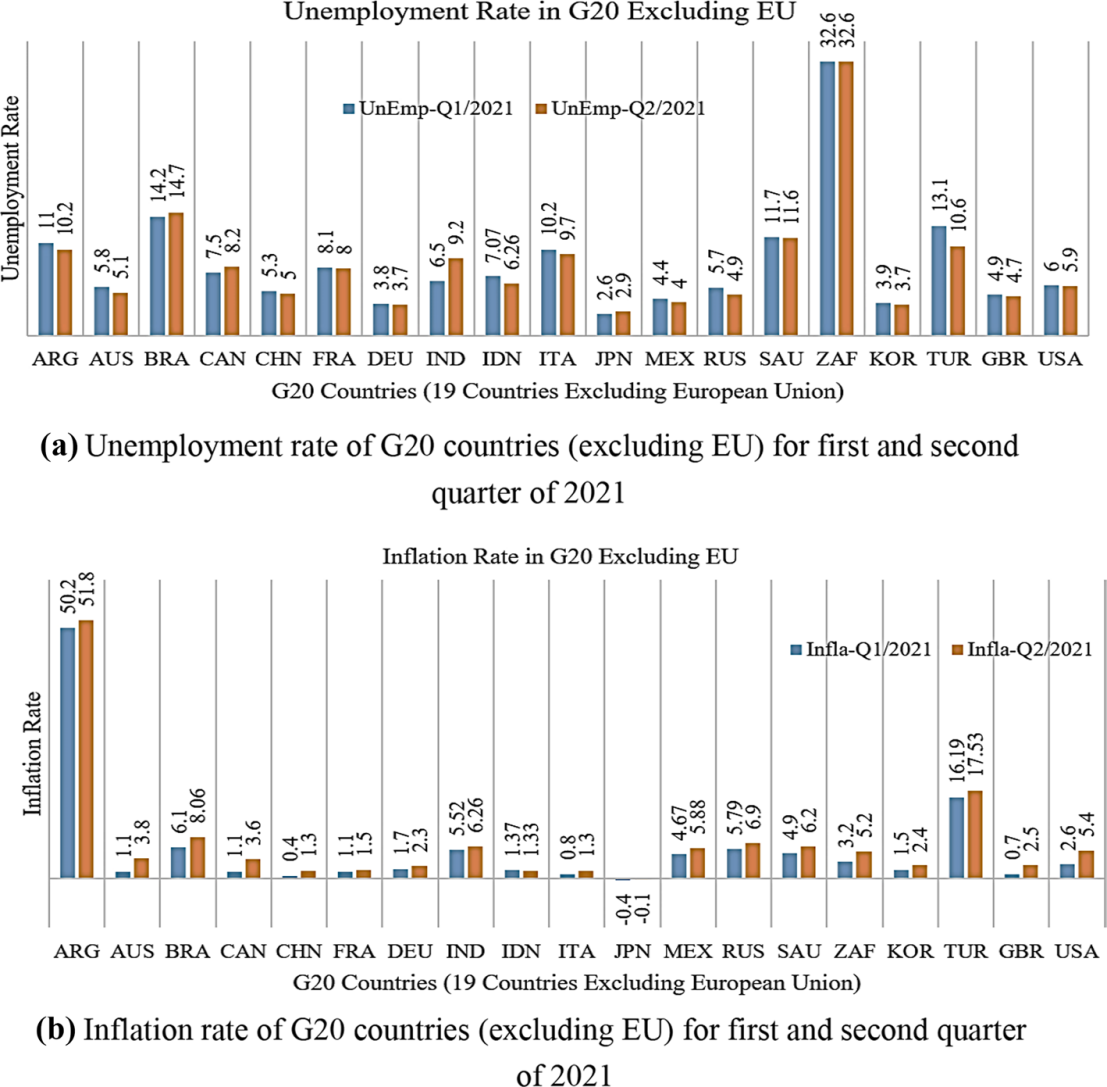
**a** Unemployment rate of G20 countries (excluding EU) for first and second quarter of 2021. **b** Inflation rate of G20 countries (excluding EU) for first and second quarter of 2021

However, emissions declined during the post-recession period, despite accelerating economic growth. COVID-19 also affected human capital growth owing to lockdown-generated disruptions in mid- and small-sized manufacturing enterprises, schooling, and training, which harmed cumulative and firm-level productivity in 2020. The G20 countries with more rigid lockdowns during 2020 experienced, on average, a more significant drop in labor market participation. Unfortunately, Fig. 11a illustrates that the unemployment problem is still in progress during the first two quarters of 2021; the highest unemployment rate appears in South Africa, Brazil, Saudi Arabia, Argentina, and Turkey at more than 10%. D’Adamo et al. (2021) claimed that although data are available only for a subset of G20 countries, the economic drop is mainly deeper for developing countries than for advanced economies due to decimated business travel and tourism and diminished movement of all stripes. Firms in advanced economies are expected to scale down investments, particularly if uncertainties regarding the COVID-19 pandemic persist. D’Adamo et al. (2021) observed that, in 2020, investment is expected to fall in all but two G20 countries, China and Turkey, compared to 2019. Comparing the effects of the COVID-19 crisis on investment with those of the global financial crisis, its influence on the G20 economies seems to be less than or comparable to that of 2009, whereas in developing market economies, it seems to be higher than average.

As seen in Fig. 11a, b, COVID-19 provides several favorable conditions to create a remarkable rate of unemployment and inflation for the periods 2020 and 2021, Q1 and Q2, around the world, especially in the G20 countries. It is crucial to point out that the post-effects of COVID-19 have been expanding exponentially worldwide, distancing global economies from potential normalization. Recently, COVID-19 data indicated a total of 211,364,677 cases, 4,423,507 deaths, 4,934,496,760 total doses administered, 1,899,999,918 fully vaccinated persons, and 188,247,177 recoveries all over the world through July 2021 (WHO 2021). Given the COVID-19 shock, the crisis is expected to leave scars, possibly through indirect mechanisms of spillover and investment declines due to hysteresis, travel bans, and fear. R&D investments are central in response to the COVID-19 pandemic to reduce unemployment and inflation rates in response to the pandemic.

In contrast, an economy’s ability to invest in innovative areas depends on country-specific characteristics including economic structure, policies, politics, institutions, and governance (Corrado et al. 2009). Looking to the future and evaluating the implications of the COVID-19 pandemic on international tax systems, many uncertainties (exogenous factors) can be observed (Baker 2020). Thus, counterplans need to be made to assess and reduce the effects of Covid-19 on economies. This may include swift and reasonably appropriate long-term plans, which may require a great deal of essential work and careful implementation, even if they are discarded or need to be significantly amended. The pandemic has had enormous and dramatically damaging economic consequences.

Moreover, the pandemic can affect prices in highly different ways; unfortunately, it has led to a considerable fall in supply and demand. For instance, policymakers in some Eurozone countries such as Belgium, Austria, and Germany adopted a VAT reduction policy that directly influenced purchase prices as another factor in reducing the economic recession and contributing to the reduction of inflation. In addition, the federal government of Germany reduced VAT by 3%, from 19 to 16% for most products and from 7 to 5%. This represented approximately 2% of the total between July and December 2020.

## Conclusions

The economic impacts of the COVID-19 pandemic are still affecting the world but are rapidly becoming a new direction for supporting development and reducing economic downturns. According to the analysis, the extended impacts of the pandemic will appear clearly in the coming years, which will help policymakers formulate policies and make decisions regarding their economic activities and financial innovations. This study addresses the first step toward filling the gap between correlated economic and health activities by delineating the clusters presented in the G20 countries. To understand how G20 countries deal with the impacts of COVID-19, this study grouped (clusters) G20 countries to understand hidden economic patterns and identify shared variables within which some countries paid more consideration than others. In identifying different groups of G20 countries, it was found that different patterns of relationships could be interesting to explore future studies in detail. This suggests that the intuition behind the clusters may be valid in some situations and can generalize the relationships, to some extent, between a group of countries. However, this generalization does not apply to any group. In addition, future studies should investigate the complex patterns of different variables more profoundly.

Moreover, this study investigated the recessionary impact of the COVID-19 pandemic on the economies of the G20 countries. Fluctuations in GDP caused stagnation or inflation due to postponed expenses and government policies related to monetary emissions. The governments of G20 countries have offered recovery plans to support the economies in different ways and have increased health expenses, supported laborers who lost their jobs, and supported mid- and small-sized enterprises to reduce unemployment and inflation as well as to recover from stagnation and recession. The current economic situation has been affecting inflation and stimulating unemployment dynamics in the G20 countries, with wide variations in economic characteristics.

This study presents several significant findings with implications for policymakers. Government spending on public health can positively impact health opportunities, which can strongly support public capital and enhance productivity growth, thereby participating in economic improvement. Policymakers must encourage enterprises to develop sensible policies that consider the magnitude of the COVID-19 pandemic and its potential devastation on social and economic consequences.

Our results and findings provide an overview of the possible effects of the pandemic on GDP, health, industrial production, unemployment, inflation, and several other essential factors. Although the slowdown in industrial productivity growth is temporary, some structural problems and policy concerns may cause an economic recession. Although industrial production growth restarted in the second quarter of 2021, its previous lower growth is associated with lower business dynamism, traveling bans, the spread of the latest variants of COVID-19 cases, and an increased divergence among the most and minor productive firms.

The governments of the G20 countries have provided efficient financial support and capital allocation to overcoming COVID-19-related problems and prevent negative economic bubbles. They have eased access to finance and liquid assets, especially relevant to overcoming the COVID-19-related crisis for firms. These supportive economic actions bring dynamism to the business environment. In addition, economic reforms are inevitable to unlock industrial productivity growth, which aim to minimize the barriers in the firm entry of business actions, achieve growth by promoting openness to trade and foreign direct investment, implement strong competition laws and policies including well-calibrated intellectual property rights, and make the labor and product markets more responsive to economic conditions, hence reducing inflation and unemployment. Promoting access to financial sources is essential for innovative and young businesspeople and companies and to provide liquidity to solve financial problems. The lack of financial alternatives and liquid sources provided by financial organizations may reduce investment opportunities and possibilities, hinder innovations, and increase unemployment and decrease economic growth.

Further studies can be applied to countries with different payer systems (single and multi) to investigate whether a multivariate correlation between the unemployment rate, tax, price inflation, health expenditures, health factors, and productivity is significant. Moreover, other variables such as the technology innovation index, green manufacturing, government systems, credit sizes, and financial innovation index can be studied for future research to investigate the capabilities of countries dealing with an economic recession.

## Data Availability

Data are contained within the manuscript and cited in the references’ section.
